# Genome-Wide Analysis and Evolutionary Perspective of the Cytokinin Dehydrogenase Gene Family in Wheat (*Triticum aestivum* L.)

**DOI:** 10.3389/fgene.2022.931659

**Published:** 2022-08-19

**Authors:** Priyanka Jain, Ankita Singh, Mir Asif Iquebal, Sarika Jaiswal, Sundeep Kumar, Dinesh Kumar, Anil Rai

**Affiliations:** ^1^ Centre for Agricultural Bioinformatics, ICAR-Indian Agricultural Statistics Research Institute, New Delhi, India; ^2^ ICAR-National Bureau of Plant Genetic Resources, New Delhi, India; ^3^ Department of Biotechnology, School of Interdisciplinary and Allied Sciences (SIAS), Central University of Haryana, Haryana, India

**Keywords:** cytokinin, gene ontology, phylogenetics, synteny, wheat

## Abstract

Cytokinin dehydrogenase (*CKX*; EC.1.5.99.12) regulates the level of cytokinin (CK) in plants and is involved in CK regulatory activities. In different plants, a small gene family encodes *CKX* proteins with varied numbers of members. These genes are expanded in the genome mainly due to segmental duplication events. Despite their biological importance, *CKX* genes in *Triticum aestivum* have yet to be studied in depth. A total of 11 *CKX* subfamilies were identified with similar gene structures, motifs, domains, cis-acting elements, and an average signal peptide of 25 amino acid length was found. Introns, ranging from one to four, were present in the coding regions at a similar interval in major *CKX* genes. Putative cis-elements such as abscisic acid, auxin, salicylic acid, and low-temperature-, drought-, and light-responsive cis-regulatory elements were found in the promoter region of majority *CKX* genes. Variation in the expression pattern of *CKX* genes were identified across different tissues in *Triticum*. Phylogenetic analysis shows that the same subfamily of *CKX* clustered into a similar clade that reflects their evolutionary relationship. We performed a genome-wide identification of *CKX* family members in the *Triticum aestivum* genome to get their chromosomal location, gene structure, cis-element, phylogeny, synteny, and tissue- and stage-specific expression along with gene ontology. This study has also elaborately described the tissue- and stage-specific expression and is the resource for further analysis of *CKX* in the regulation of biotic and abiotic stress resistance, growth, and development in *Triticum* and other cereals to endeavor for higher production and proper management.

## Introduction

Wheat (*Triticum aestivum L*.) is a widely cultivated cereal grain that provides approximately 20% of the protein in human diet. It ranks second to corn and rice as the most important staple food, with global production of around 778.6 million tonnes of grain (USDA, 2022). Looking at the increasing human population, its consumption is estimated to rise to 900 million tonnes by 2050 (FAO, 2022). Cytokinins, the plant hormone family, affect crop yield in terms of grain size and number, and are involved in the regulation of biotic and abiotic stress (Cortleven et al., 2019). They are reported to control both shoot and root architecture as well as crown formation (Chen L. et al., 2020). Cytokinin dehydrogenase (*CKX*) gene family members encode the enzyme *CKX*, and play an important role in the cell cycle, developmental processes, and senescence process of plants such as seed germination, shoot/root growth, photosynthesis, chloroplast formation, and crop productivity (Mok and Mok 2001; Takei et al., 2001; Werner et al., 2001). Recent research suggests that a majority of plants had cytokinin (CK) metabolic genes closely linked to them and, therefore, strongly regulate cytokinin content in different organs of plants. (Ashikari et al., 2005; Bartrina et al., 2011; Yuldashev et al., 2012). Further, CKs are adenine derivatives with a side chain at the N6 position, which causes the N6 substituent to be classified as an isoprenoid or aromatic side chain (Galuszka et al., 2000; Frebort et al., 2011). Since numerous changes have been observed in the CK content during grain development in crops like wheat and barley, genetic manipulation of the genes involved in CK homeostasis is frequently utilized for yield improvement in these crops (Schmuelling et al., 2003).

*CKX* (EC: 1.5.99.12) is the only known enzyme that permanently degrades CKs by cleaving the N6-unsaturated side chain of the CK to adenine and adenosine in a single step. The catalytic activity of *CKX* is catalyzed by two conserved domains, *namely,* a FAD-binding domain at the N terminus and a CK-binding domain at the C terminus of the protein. *CKX* activity was first discovered in crude extracts from tobacco plants (Paces et al., 1971). Subsequently, *CKX* was recognized as a member of a small gene family and several *CKX* genes were duplicated and identified in different plants. Cytokinin dehydrogenase has been identified as a significant enzyme present in different cereal crops. It has been reported in 13 gene families in *Zea mays* (Gu et al., 2010; Houba-Hérin et al., 1999; Brugière et al., 2003), 11 gene families in rice (Ashikari et al., 2005), 9 gene families in Arabidopsis (Galuszka et al., 2007), 13 gene families in *Brachypodium distachyon* (Mameaux et al., 2012), four gene families in *Hordeum vulgare* (Mameaux et al., 2012), and 11–14 gene families in wheat (Ogonowska et al., 2019; Shoaib et al., 2019; Song et al., 2012). In rice, an increase in CK is a product of low Os*CKX*2 expression which promotes the total yield by increasing the number of reproductive organs using short hairpin RNA-mediated silencing technology. This hinders the expression of Os*CKX*2 in rice, resulting in an increased number of tillers, thus increasing the grain weight (Ashikari et al., 2005; Yeh et al., 2015). Similarly, *Arabidopsis CKX* genes showed diverse countenance, which supports that differential expression of *CKX* genes can play a vital role in regulating CK levels (Bartrina et al., 2011).

The CKX gene family has been reported to show natural variations in the form of SNPs which were used for the potential marker-assisted selection for higher yield as in soybean (Nguyen et al., 2021). Besides, CKs are widely used for crop yield improvement/management because of their broader effects on plant growth/development and physiology. Applications of CRISPR-based gene-editing tools in manipulating cytokinin metabolism at the genetic level for yield improvement has been reported in literature (Mandal et al., 2022). Genome editing occurs via natural process of random mutagenesis, mostly through SNPs to improve yield (Songstad et al., 2017).

Although > 20 different cytokinins are reported in wheat (Sýkorová et al., 2008), the complete analysis on the early development time frame has not yet been reported for wheat. The hexaploidy, large genome size (∼17 GB), and complicated interactions between the three genomes of wheat (*Triticum aestivum* L.) have led to limited information on the CKX gene families in this cereal crop.

This study aims at studying the genome-wide identification and distribution of *CKX* genes in wheat, motif characteristics, distribution and evolutionary relationships along with the presence of cis-elements in the promoter regions across 11 subfamily members of CKX. This study also aims at comparing motifs and domains present among the CKX family members at the protein level along with tissue- and stage-specific expression. This analysis will lead to useful information for further functional dissection of *CKX* genes in plants.

## Materials and Methods

### Identification of *CKX* Family Members in *Triticum*

The *CKX* genes were identified from the *Triticum aestivum* (bread wheat) genome (IWGSC RefSeq), available at Ensemble plant database (https://plants.ensembl.org/index.html). The cytokinin dehydrogenase (*CKX*), FAD- and cytokinin-binding (cytokinin-bind PF09265) protein domain, family was obtained from the PFAM database (https://pfam.xfam.org/). HMMER 3.0 program was used to identify the *CKX* protein domain-containing putative protein in bread wheat. Blastp was performed on these proteins with parameters e < 1e− 5 and 50% identity, keeping the query as *Oryza sativa, Arabidopsis thaliana, Hordeum vulgare, and Zea mays* to find the putative *CKX* protein from the bread wheat protein sequence. The NCBI-CDD web server (https://www.ncbi.nlm.nih.gov/Structure/bwrpsb/bwrpsb.cgi) was used to confirm the candidate *CKX* genes in *Triticum aestivum*. ProtComp was used to predict the subcellular localization (Kamper et al., 2006) followed by the identification of the signal peptides and the location of their cleavage sites in *CKX* protein using SignalP (Armenteros et al., 2019). TargetP was used to identify the presence of N-terminal presequences: signal peptide (SP), mitochondrial transit peptide (MTP), chloroplast transit peptide (cTP), or thylakoid luminal transit peptide (lTP) in *CKX* proteins. Other protein parameters like molecular weight (MW) and theoretical isoelectric point (PI) of *CKX* proteins was found using the ExPASy server (Gasteiger et al., 2003). The subcellular localization predictions of the analyzed proteins were also performed using WoLF PSORT (https://wolfpsort.hgc.jp/).

### Chromosomal Distribution, Gene Structure, and Conserved Motif of *CKX* Gene Family

The distribution of *CKX* genes was observed with its chromosomal location using Plant ensemble database (Bolser et al., 2016). Also, their gene structures were displayed in the Gene Structure Display Server (GSDS: http://gsds.gao-lab.org/; Guo et al., 2007). Motif identification was performed using the MEME suite. The multiple expectation maximization for the motif elicitation with MEME v4.9. (Bailey and Elkan, 1994) utility program (http://meme-suite.org/tools/meme) was used to display motifs in *CKX* proteins.

### Phylogenetic and Synteny Analysis of the *CKX* Gene Family

Multiple sequence alignment was performed through ClustalW (Thompson et al., 1994) based on the full sequence of the proteins with default parameters from *Triticum aestivum*, *Oryza sativa, Arabidopsis thaliana, Hordeum vulgare, and Zea mays.* The phylogenetic tree was constructed by maximum likelihood method using the MEGA11 (Tamura et al., 2021) software with 1,000 replicates as bootstrap values. iTOL (https://itol.embl.de/) was used to visualize the phylogenetic tree and motifs in the *CKX* proteins (Letunic and Bork, 2019).

TBtool (Chen et al., 2020b) (https://github.com/CJ-Chen/TBtools/releases) was used for synteny analysis of *CKX* genes in *Triticum aestivum* with *Aegilops tauschii* via the MCScanX function. MCScanX is a toolkit for the detection and evolutionary analysis of gene collinearity (Wang et al., 2012).

### Promoter and Gene Ontology Analysis of *CKX* Gene Family With Expression Across Different Tissue and Stages

A 1.5-kb region upstream from the start of the gene was retrieved for the promoter analysis from the *CKX* gene. The PlantCARE (Lescot et al., 2002) database was used for the identification of cis-regulatory elements in the promoter region. *CKX* genes in developmental and tissue time course expression in *Triticum* varieties were obtained from the wheat expression browser (http://www.wheat-expression.com/download) to detect the expression profile of the *CKX* gene. Gene ontology analysis was performed using the PlantRegMap tool (Tian et al., 2020).

## Results and Discussion

### Identification of the *CKX* Gene Family in *Triticum aestivum*

*CKX* proteins were identified from the whole genome of *Triticum aestivum* using blastp and HMM. The putative *CKX* proteins were confirmed using Pfam and a conserved domain database. In earlier published literature, 11–14 gene family members have been reported in wheat (Ogonowska et al., 2019; Shoaib et al., 2019). In rice, 11 *CKX* homologs have been identified and in *Arabidopsis thaliana*, 7 homologs of the *CKX* gene, in *Hordeum vulgare* 15, and in *Zea mays* 34 homologs of *CKX* were identified.

Earlier studies on *CKX* have given different nomenclatures to the different gene families of *CKX*. We have renamed *CKX* based on recent nomenclature in IWGSC taking into consideration the earlier reports and true orthologs in rice and maize. Finally, 11 *CKX* families were identified and their genomic location was extracted from Plant Ensemble. The average molecular weight (MW) and theoretical isoelectric point (PI) of *CKX* proteins ranged from 50,000 to 60,000 Da and from 5‐8, respectively (Table 1).

**TABLE 1:** Detailed information of the cytokinin dehydrogenase gene family (*Triticum aestivum*) with respect to their chromosomal location and general protein parameters.

Suggested Nomenclature	Gene ID	Protein Stable ID	Chromosome	Gene Start (bp)	Gene End (bp)	Gene Length	Protein Length	No. of Exons	Theoretical pI	Molecular Weight (Average) Dalton (Da)
CKX1_3A	TraesCS3A02G109500	TraesCS3A02G109500.1	3A	75545535	75547971	2436	524	3	5.59	56150.7
CKX1_3B	TraesCS3B02G128700	TraesCS3B02G128700.1	3B	107958655	107961101	2446	524	3	5.75	56149.79
CKX1_3D	TraesCS3D02G111300	TraesCS3D02G111300.1	3D	64761714	64764211	2497	524	3	5.78	56112.69
CKX2.1_3A	TraesCS3A02G311000	TraesCS3A02G311000.1	3A	549902478	549907314	4836	567	3	7.16	61590.05
CKX2.1_3B	TraesCS3B02G161100	TraesCS3B02G161100.1	3B	157763715	157768151	4436	578	3	5.81	62557.13
CKX2.1_3D	TraesCS3D02G143600	TraesCS3D02G143600.1	3D	107041806	107046044	4238	551	3	6.17	59559.72
CKX2.2.1_3A	TraesCS3A02G311100	TraesCS3A02G311100.1	3A	550047133	550050899	3766	552	3	5.56	59371.38
CKX2.2.1_3B	TraesCS3B02G161000	TraesCS3B02G161000.1	3B	157660250	157664488	4238	547	3	5.56	59014.96
CKX2.2.1_3D	TraesCS3D02G143500	TraesCS3D02G143500.1	3D	106736568	106740664	4096	547	3	5.57	59158.15
CKX2.2.2_3D	TraesCS3D02G143300	TraesCS3D02G143300.1	3D	105891267	105895512	4245	547	3	5.65	59195.27
CKX2.2.3_3D	TraesCS3D02G143200	TraesCS3D02G143200.1	3D	105704626	105709481	4855	523	3	5.24	56059.49
CKX3_1A	TraesCS1A02G159600	TraesCS1A02G159600.1	1A	286006369	286010711	4342	522	5	6.09	57687.03
CKX3_1B	TraesCS1B02G176000	TraesCS1B02G176000.2	1B	317439232	317444264	5032	522	5	6.28	57740.1
CKX3_1D	TraesCS1D02G157000	TraesCS1D02G157000.1	1D	221120218	221124390	4172	522	5	6.17	57673.02
CKX4_3B	TraesCS3B02G525300	TraesCS3B02G525300.1	3B	766978875	766981579	2704	525	5	6.57	57557.89
CKX4_3D	TraesCS3D02G475800	TraesCS3D02G475800.1	3D	576755407	576758217	2810	525	5	6.66	57540.78
CKX5_3A	TraesCS3A02G321100	TraesCS3A02G321100.1	3A	564437512	564442094	4582	530	5	5.94	57743.55
CKX5_3B	TraesCS3B02G344600	TraesCS3B02G344600.1	3B	554115957	554120243	4286	531	5	6.15	57876.68
CKX5_3D	TraesCS3D02G310200	TraesCS3D02G310200.1	3D	424349539	424353886	4347	531	5	6.03	57823.56
CKX7_6B	TraesCS6B02G214700	TraesCS6B02G214700.1	6B	290213802	290215403	1,601	533	1	8.23	58047.33
CKX7_6D	TraesCS6D02G172900	TraesCS6D02G172900.1	6D	159787916	159792494	4578	467	2	6.08	50655.82
CKX8_2A	TraesCS2A02G378300	TraesCS2A02G378300.1	2A	621182540	621186230	3690	528	5	5.62	57186.26
CKX8_2B	TraesCS2B02G395200	TraesCS2B02G395200.1	2B	560261181	560265014	3833	523	5	6.32	56987.2
CKX9_1B	TraesCS1B02G248700	TraesCS1B02G248700.1	1B	439845400	439847579	2179	521	5	6.68	58242.54
CKX9_1D	TraesCS1D02G237200	TraesCS1D02G237200.1	1D	326285642	326287883	2241	521	5	6.86	58326.58
CKX10_7A	TraesCS7A02G363400	TraesCS7A02G363400.1	7A	538150224	538152325	2101	551	2	6.22	59913.79
CKX10_7B	TraesCS7B02G264400	TraesCS7B02G264400.1	7B	484892227	484894256	2029	540	2	6.19	59024.94
CKX10_7D	TraesCS7D02G359700	TraesCS7D02G359700.1	7D	461956360	461958099	1739	532	2	6.15	57595.18
CKX11_7A	TraesCS7A02G536900	TraesCS7A02G536900.1	7A	714340482	714342889	2407	516	4	5.77	55390.7
CKX11_7B	TraesCS7B02G455000	TraesCS7B02G455000.1	7B	715713483	715716700	3217	516	4	5.93	55654.04
CKX11_7D	TraesCSU02G106300	TraesCSU02G106300.1	Un	93131788	93134766	2978	517	4	5.99	55628.01

**FIGURE 1:**
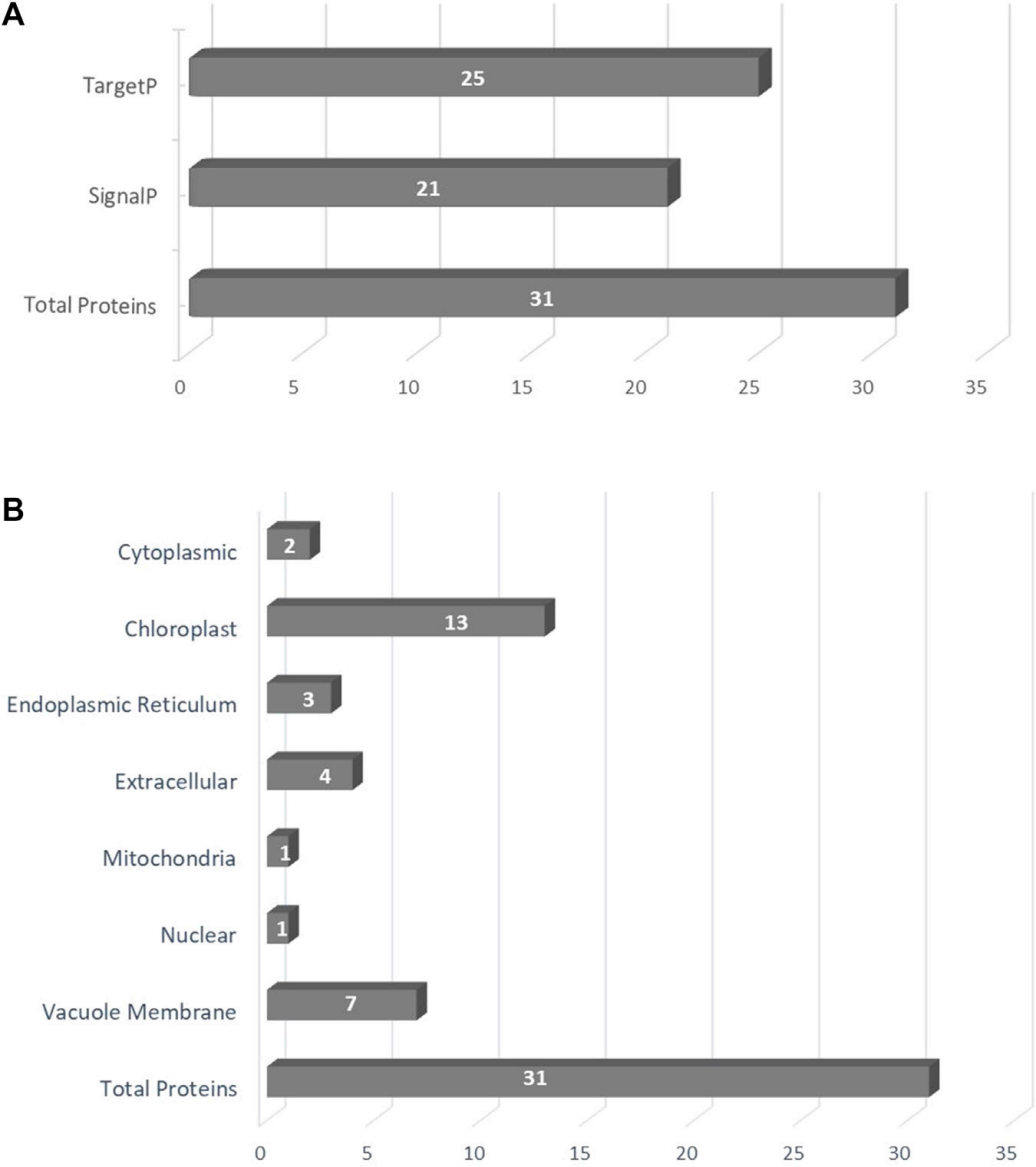
**(A)** The subcellular localization of predicted proteins; **(B)** The presence of signal peptides in the analyzed sequences using SignalP and TargetP.

To determine whether the analyzed proteins are directed to the secretory pathway and secreted out of the cell, predictions of the presence of signal peptides at the N terminus of the sequence were performed. For this purpose, program SignalP-6.0 and TargetP were used. Using the SignalP, the signal peptides were found in 21 of the analyzed proteins. The average length of sequences provided as a signal peptide was 25 aa. Similar results were obtained using the TargetP tool—the signal peptides were found in 25 of the investigated sequences (Figure 1A; Supplementary Tables S1A,B). The most likely location of the *CKX* protein was predicted as extracellular using the ProtComp tool. The subcellular localization prediction program, WoLF PSORT helps to trace the protein in the intracellular compartments. Out of the total analyzed proteins, 13 sequences were classified as chloroplast, 7 as the vacuole membrane protein, 4 as extracellular, 3 as endoplasmic reticulum, 2 as cytoplasmic, 1 as the mitochondria, and 1 as the nuclear pathway (Figure 1B).

### Chromosomal Distribution, Gene Structure, and Conserved Motif of *CKX* Genes

**FIGURE 2:**
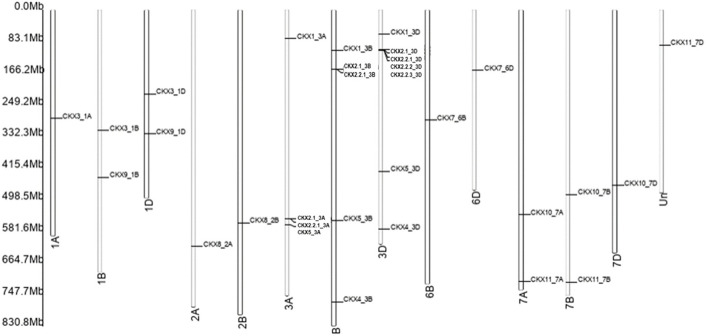
Chromosomal locations of the CKX gene family in *Triticum aestivum*.

**FIGURE 3:**
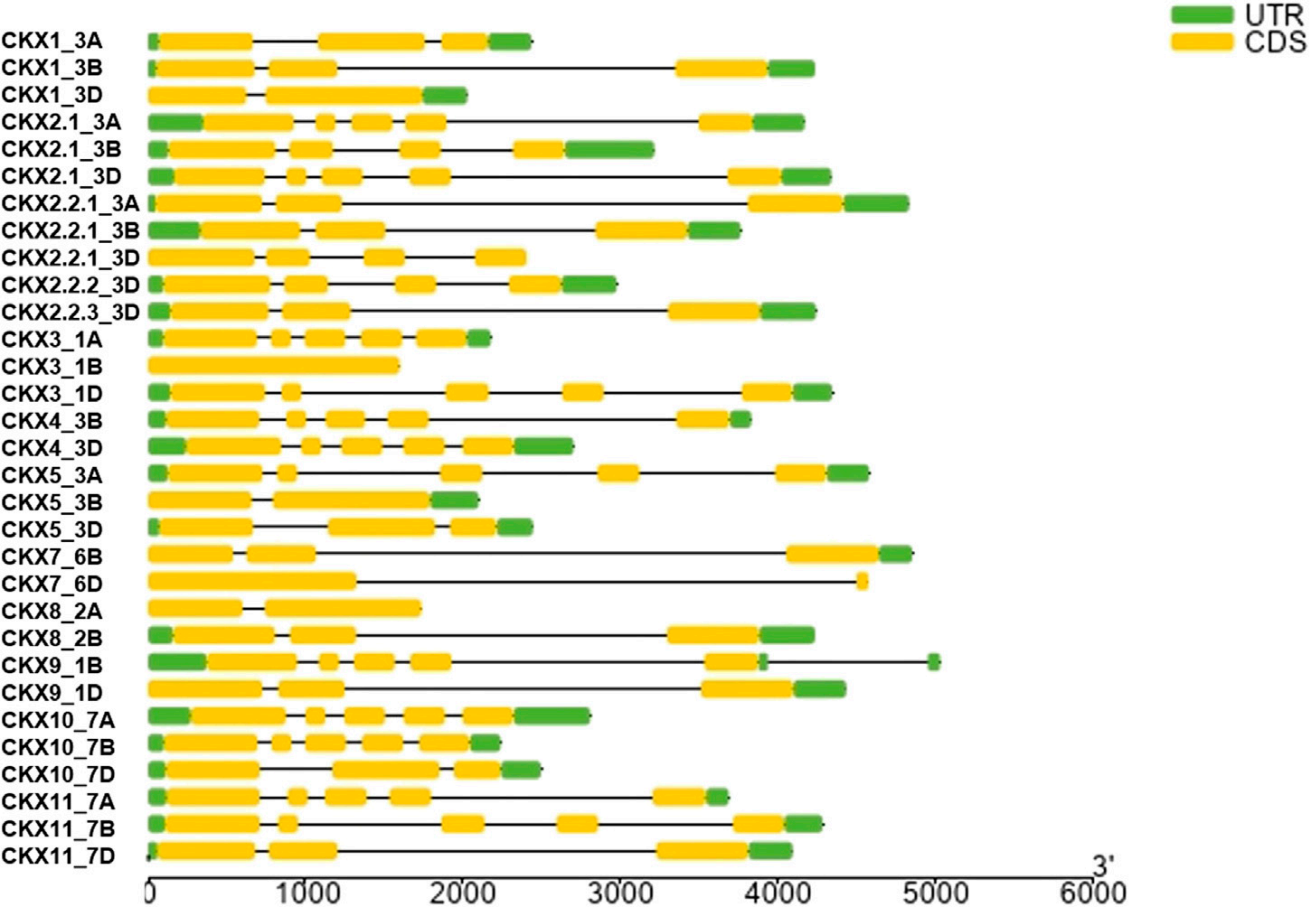
Gene structures of the CKX gene family in *Triticum aestivum*.

Motif diversity and gene structure promote evolution in gene families (Zhu et al., 2020). We identified 31 Ta*CKX* gene family members that were present on chromosomes, linked with the A, B, and D subgenomes of *Triticum*. Among the 11 gene families, Ta*CKX*2 has gene duplication on chromosome 3D, that is further subdivided, based on homology, into different groups (Mameaux et al., 2012; Lu et al., 2015). *CKX* gene families identified different homologs in the subgenome A, B, and D. All *CKX* gene family members were found on chromosomes 1 to 7, except chromosome 6 (Figure 2). One of the gene families, *i.e.,* the *CKX1*1 subfamily was not present on any of the 21 chromosomes (of A, B, and D subgenomes) in IWGSC Refseq. In the *CKX* genes, a number of exons varied from 1 to 5. Only *CKX7*_6B identified one exon and no intron while 11 *CKX* genes and 13 *CKX* genes had 3 exons and 5 exons in their gene structures, respectively (Figure 3). The gene transfer format (gff) was used to extract the 5′ UTR, 3′ UTR, exonic, intronic, start, and end position of the gene. It was found that the 5’ UTR position was absent for *CKX2.1_3B, CKX2.2.3_3D, CKX7_6B, CKX7_6D, CKX10_7A, CKX11_7A, CKX10_7B,* and *CKX1*0_7D gene family members in the gff file. Intronic region regulated region expression via small and long noncoding RNA in genome (Jain et al., 2021). *CKX* gene structures showed that a majority of the genes had the presence of more than one intron. However, the length of the intron kept varying between the subgroups. The longest intron was found in *CKX*7_6D. Previous studies show that introns present in *β*-tubulin might regulate the expression and evolution of genes in *Fusarium graminearum* (Li et al., 2019).

Comparison of 31 *CKX* family members showed the presence of the PLN02441 domain family. The predicted *CKX* proteins had typical FAD- and CK-binding domains, which were specific to *CKX* family members as shown in Supplementary Figure S1. Similar results have been obtained in genome-wide analysis and identification of cytokinin oxidase/dehydrogenase (*CKX*) gene family in foxtail millet (Liu et al., 2018).

### Phylogenetic and Synteny Analysis of the *CKX* Gene Family

**FIGURE 4:**
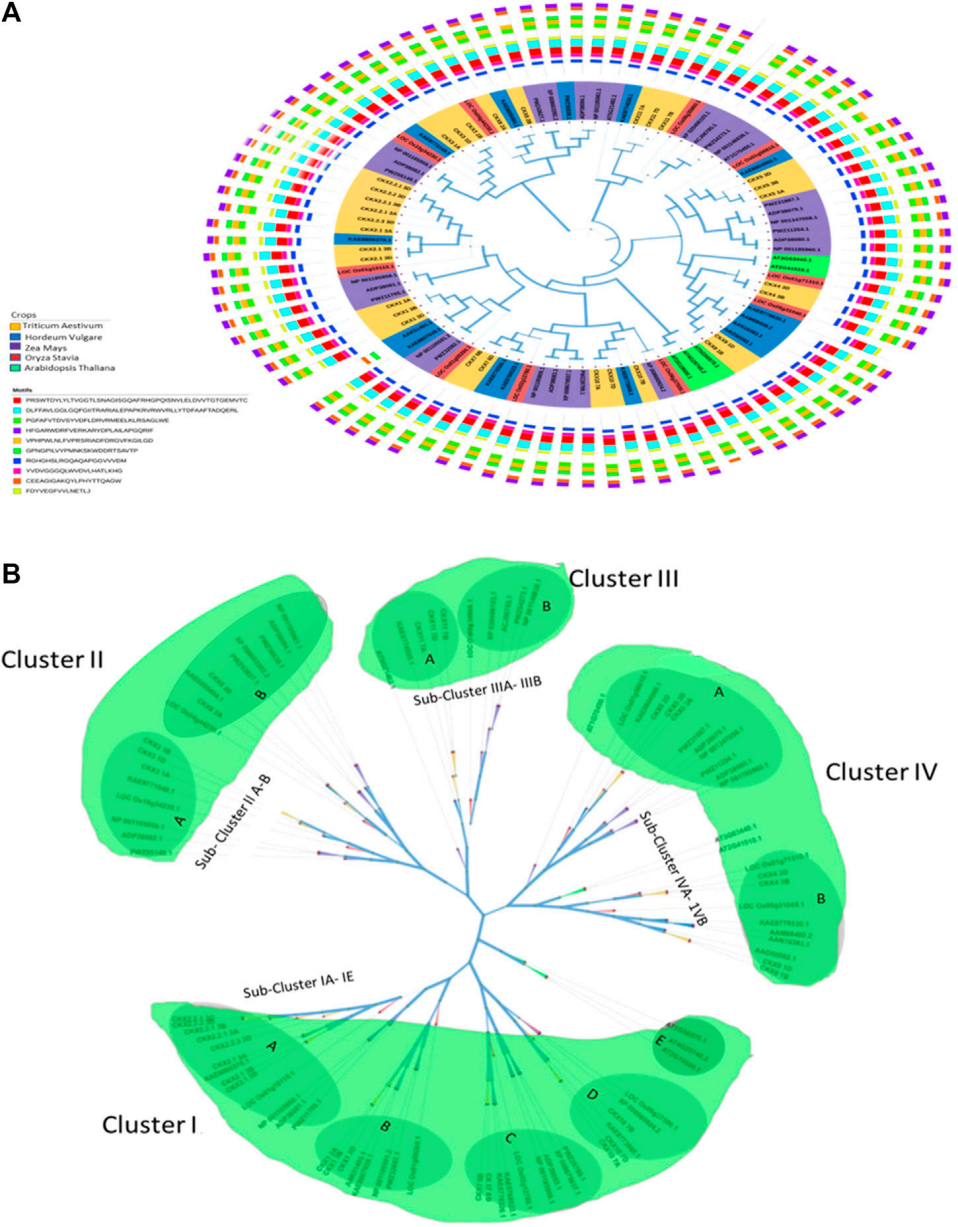
**(A)** Phylogenetic tree for CKX proteins of *Triticum aestivum*, *Oryza sativa, Arabidopsis thaliana, Hordeum vulgare,* and *Zea mays*. **(B)**: Four major clusters of CKX proteins of *Triticum aestivum, Oryza sativa, Arabidopsis thaliana, Hordeum vulgare*, and *Zea mays* using the maximum likelihood method.

Highly conserved motif regions in *CKX* proteins were identified using MEGA 11 in classic mode with the maximum number of motifs set at ten. Ten-motif consensus sequences were identified in all 31 *CKX* proteins as shown in Figure 4A. To uncover the evolutionary relationships among *Triticum aestivum*, *Oryza sativa, Arabidopsis thaliana, Hordeum vulgare,* and *Zea mays CKX*s, the amino acid sequences of the *CKX* genes were aligned using ClustalW. This was followed by the maximum likelihood analysis method to construct a phylogenetic tree (Figures 4A,B). All the proteins fell into four major clusters (I, II, III, and IV). Cluster I contained 39 members (with 16 of *Triticum aestivum*, 1 *Oryza sativa*,1 *Hordeum vulgare*, 3 *Zea mays,* and 3 *Arabidopsis thaliana,* respectively). This could be further divided into the following subclusters: IA,IB,1C,1D, 1E, and a diverge member (*CKX*). Cluster II included 18 members (with five of *Triticum aestivum*, two *Oryza sativa*, two *Hordeum vulgare,* and nine *Zea mays*) and could be separated into subclusters IIA and IIB. Cluster III contained 11 members (three *Triticum aestivum*, one *Oryza sativa*, one *Hordeum vulgare*, and five *Zea mays*) that could be separated into subclusters IIIA and IIIB. Cluster IV had 30 members (with seven *Triticum aestivum*, three *Oryza sativa*, five *Hordeum vulgare,* six *Zea mays,* and two *Arabidopsis thaliana,* respectively) which could be further divided into subclusters IVA and IVB.

The paralogs have been separated into two subgene families (*CKX2.1* and *CKX2*.2) as shown in the phylogenetic tree (Figure 4B). Notably, KAE8806376.1 and LOC_Os01g10110.1 from *Hordeum vulgare* and *Oryza sativa*, respectively grouped with the *CKX2.1* and *CKX2.2, CKX1* was grouped with AAK51495.1 and KAE8807039.1 (*Hordeum vulgare*) and *CKX7* with KAE8776326.1 and KAE8768522.1 (*Hordeum vulgare*) subclusters, respectively. Similarly, Cluster II *CKX3* 1A was grouped with KAE8771049.1 and LOC_Os10g34230.1 (*Oryza sativa*) and *CKX8* 2A were grouped with KAE8805484.1 (*Hordeum vulgare*) while *CKX8* 2B was grouped with LOC_Os04g44230.1 (*Oryza sativa*). In Cluster III, *CKX1*1 was clustered with KAE8774039.1 (*Hordeum vulgare*) and in Cluster IV, *CKX5* 3D was clustered with KAE8804960.1 and *CKX7* 3D LOC_Os01g71310.1. Functional investigation of these new members is critical as they could be of considerable importance for genetic improvement.

**FIGURE 5:**
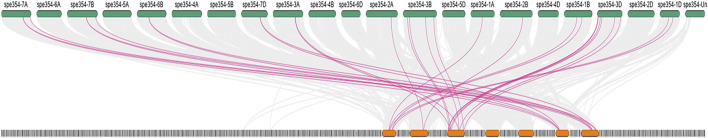
Synteny and collinearity analysis between *Triticum aestivum* and *Aegilops tauschii*.

Gene duplication, an indispensable mechanism, can expand new genes that share similar or different functions (Ohno., 1970). Therefore, we analyzed the duplication events that occurred in the gene family through synteny and collinearity of the *CKX*S gene. Three *CKX2* have undergone gene duplication suggesting that the *CKX2s* on chromosome 3D could be subdivided into two groups based on their homology. Collinearity is identified between *Triticum aestivum* and *Aegilops tauschii* as shown in (Figure 5; Supplementary Table S2). Similar results for synteny has been identified in *Brassica oleracea* and *Arabidopsis thaliana CKX* genes (Zhu et al., 2022)

### Promoter and Gene Ontology Analysis of *CKX* Gene Family

**FIGURE 6:**
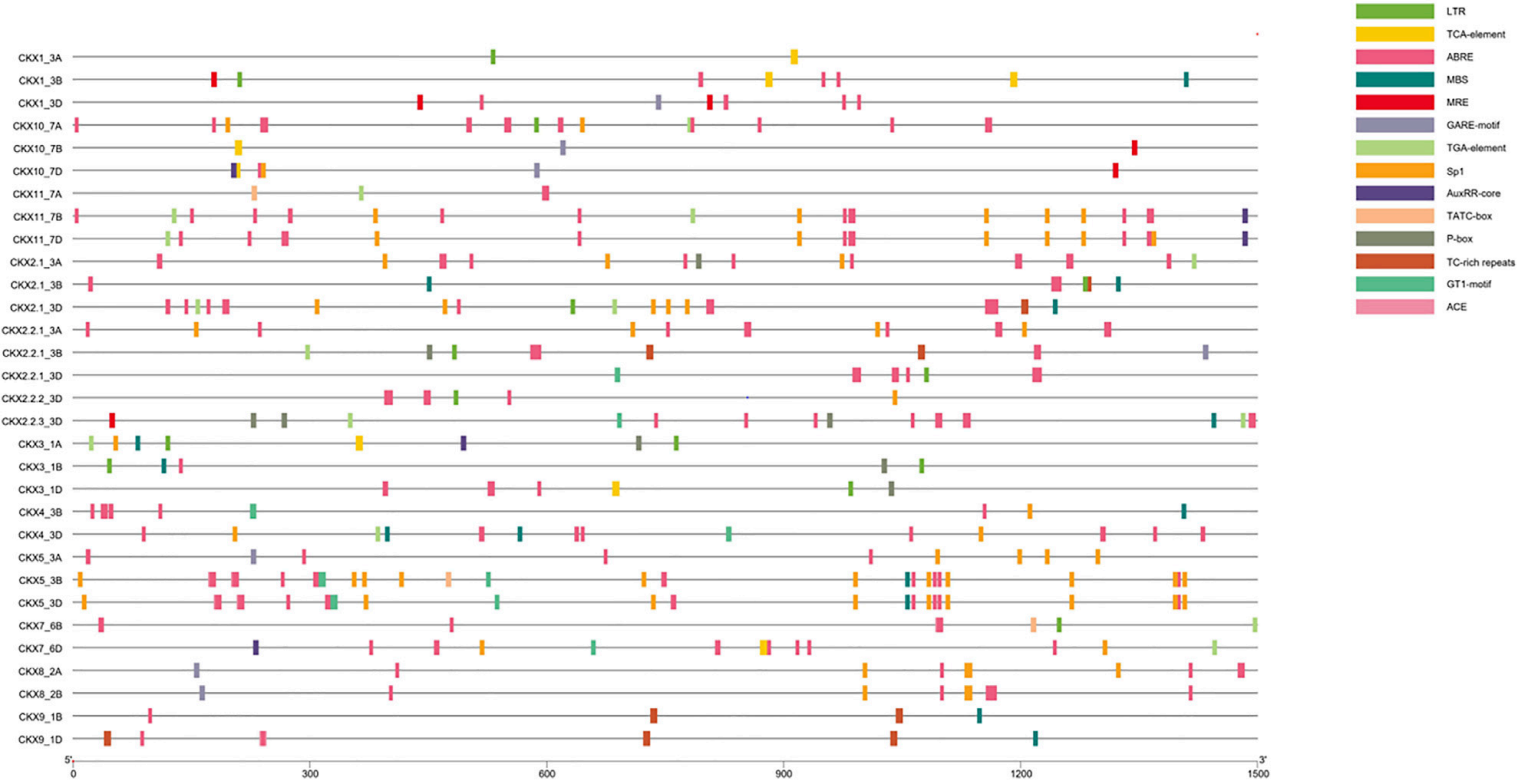
Promoter analysis of CKX gene family in *Triticum aestivum*.

*Cis*-regulatory elements present in the promoter region play an important role in regulating gene expression of metabolic pathway-related genes (Lescot et al., 2002). In a 1.5-kb upstream region of *CKX* family members, the cis-regulatory elements predicted were mainly phytohormone responsive (salicylic acid, abscisic acid, gibberellin, auxin-responsive element), drought, light, defense, and stress responsive (Figure 6; Supplementary Table S3). Cis-acting element involved in low-temperature responsiveness (LTR) was found in *CKX1_3A, CKX1_3B, CKX2.1_3B, CKX2.1_3D, CKX2.2.1_3B, CKX2.2.1_3D, CKX2.2.2_3D, CKX3_1A,CKX3_1B,CKX3_1D, CKX7_6B,* and *CKX1-_7A.* Transcription factor MYB-binding site involved in drought inducibility (MBS) was found in *CKX1_3B, CKX2.1_3B, CKX2.1_3D, CKX2.2.3_3D, CKX3_1A, CKX3_1B, CKX4_3B, CKX4_3D, CKX5_3B, CKX5_3D, CKX9_1B,* and *CKX9_1D.* The MYB-binding site involved in light responsiveness (MRE) was found in *CKX1_3B, CKX1_3D, CKX2.2.3_3D, CKX1-_7B,* and *CKX1-_7D*. Light responsive element (*Sp1*) was found in *CKX2.1_3A, CKX2.1_3D, CKX2.2.1_3A, CKX2.2.2_3D, CKX3_1A, CKX4_3B, CKX4_3D, CKX5_3A, CKX5_3B, CKX5_3D, CKX7_6D, CKX8_2A, CKX8_2B, CKX1-_7A, CKX1-_7D, CKX11_7B,* and *CKX11_7D.* Light responsive element (GT1-motif) was found in *CKX2.2.1_3D, CKX2.2.3_3D, CKX4_3B, CKX4_3D, CKX5_3B, CKX5_3D,* and *CKX*7_6D. Cis-acting element involved in light responsiveness (ACE) was observed in *CKX*5_3B, *CKX5_3D, CKX7_6B,* and *CKX9_1D*. Cis-acting element involved in defense and stress responsiveness (TC-rich repeat) was found in *CKX2.1_3B, CKX2.1_3D, CKX2.2.1_3B, CKX9_1B,* and *CKX9_1D*. In tomato TC-rich repeats were found in seven promoters of protein disulfide isomerases (PDI) and high expression of PDI was found in response to abiotic stress in tomato (Wai et al., 2021). Cis-acting element involved in salicylic acid responsiveness (TCA element) was found in *CKX1_3A, CKX1_3B, CKX2.1_3A, CKX3_1A, CKX3_1D, CKX7_6D, CKX1_7B,* and *CKX1-_7D.* Cis-acting element involved in the abscisic acid responsiveness (ABRE) was found in *CKX1_3B, CKX1_3D, CKX2.1_3A, CKX2.1_3B, CKX2.1_3D, CKX2.2.1_3A, CKX2.2.1_3B, CKX2.2.1_3D, CKX2.2.2_3D, CKX2.2.3_3D, CKX3_1B, CKX3_1D, CKX4_3B, CKX4_3D, CKX5_3A, CKX5_3B, CKX5_3D, CKX7_6B, CKX7_6D, CKX8_2A, CKX8_2B, CKX9_1B, CKX9_1D, CKX1-_7A, CKX1-_7D, CKX11_7B,* and *CKX11_7D.* Gibberellin-responsive element (GARE-motif) was found in *CKX1_3D, CKX2.2.1_3B, CKX5_3A, CKX8_2A, CKX8_2B, CKX1-_7B, and CKX1-_7D.* Auxin-responsive element (TGA-element) was *found in CKX2.1_3D, CKX2.2.1_3B, CKX2.2.3_3D, CKX3_1A, CKX4_3D, CKX7_6B, CKX7_6D, CKX1-_7A, CKX11_7B,* and *CKX11_7D.* Cis-acting regulatory element involved in auxin responsiveness (AuxRR-core) was found in *CKX3_1A, CKX7_6D, CKX1-_7D, CKX11_7B,* and *CKX11_7D.* Cis-acting element involved in gibberellin responsiveness (*TATC-box*) was found in *CKX5_3B* and *CKX7_6B.* Gibberellin-responsive element (*P-box*) was found in *CKX2.1_3A, CKX2.2.1_3B, CKX2.2.3_3D, CKX3_1A, CKX3_1B,* and *CKX3_1D*. In *Brassica napus, the CKX* gene family also has abscisic acid-responsive elements (ABRE), auxin-responsive elements (TGA-element), gibberellin-responsive elements (GARE-motif, P-box, and TATC-box), and salicylic acid-responsive elements (TCA-element)(Liu et al., 2018). Phytohormone-responsive cis-elements were found in majority of the promoter region of *CKX* genes, reflecting that cytokinins are involved in the development and growth of the plant. Cis-acting element involved in the abscisic acid responsiveness (ABRE) was found in the highest number (27) among all responsive elements. Literature supports the cross-talk between cytokinin and abscisic acid (El-Showk et al., 2013).

### Validation of CKX Gene Family’s Expression Across Different Tissues of Wheat

Cytokinin receptor kinases (AHK2 and AHK3) inhibit ABA signaling for regulating cold stress response (Jeon et al., 2010). Hence, *CKX* and ABA crosstalk to regulate the stress responses. It has been validated earlier using qRT-PCR that ABA downregulates *CKX* genes (Werner et al., 2006). *CKX5_3B, a* family member of *CKX,* has the highest number of cis-elements.

**FIGURE 7:**
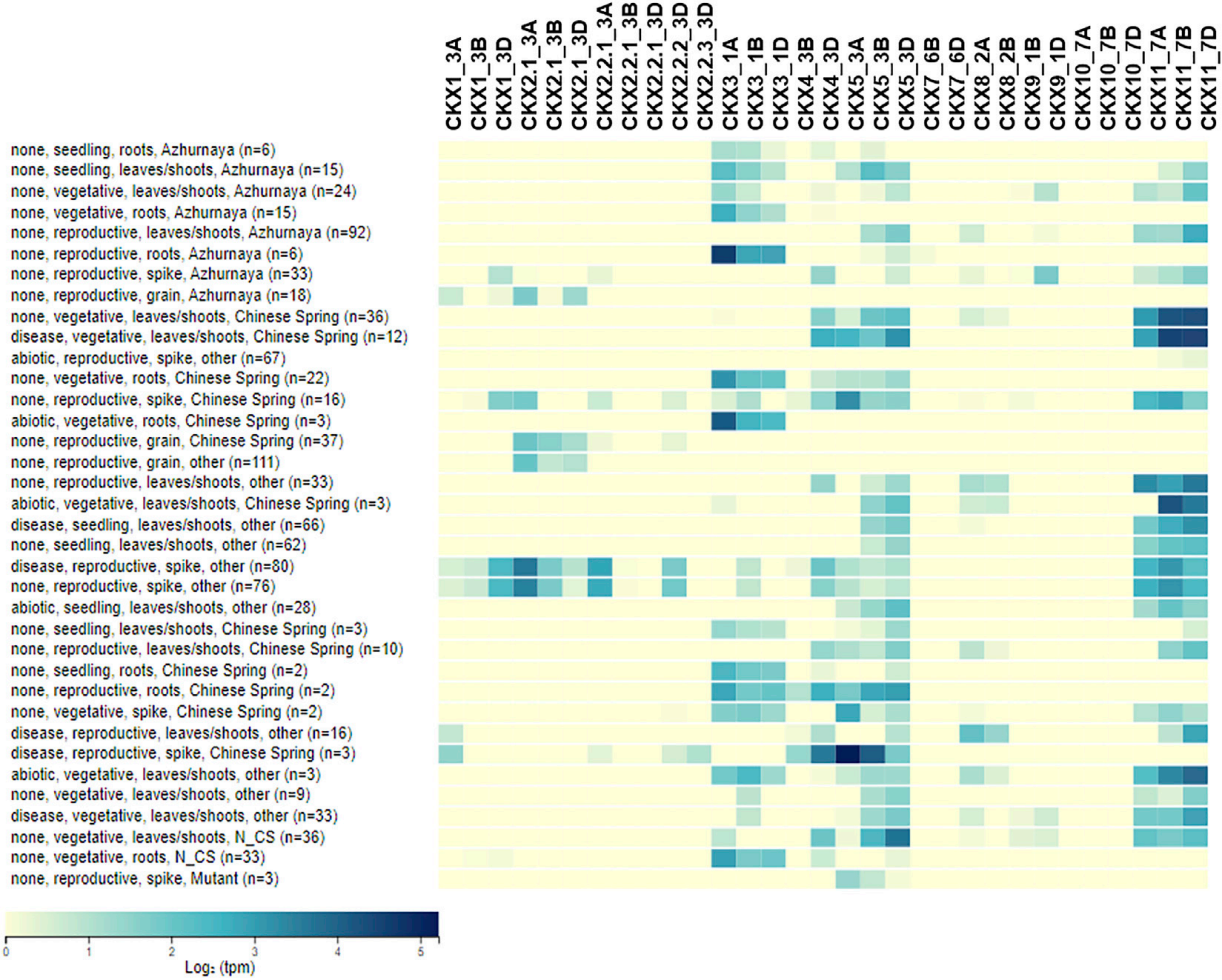
Heatmap for gene expression trend of CKX gene family in different tissue and stages of *Triticum aestivum*.

The different regulatory elements predicted responsive to abiotic and biotic stress show that the *CKX* family is stabilizing CK content under both abiotic and biotic stresses. Gene expression pattern helps to identify the key genes playing a role in an important biological function (Jain et al., 2017; Jain et al., 2019). *CKX* gene family members in our study show differential tissue-specific expression (Figure 7). Expression of GmCKX03, 05, 10, and 11 was reported to be low while GmCKX13, 15, and 16 were highly expressed in soybean (Le et al., 2012). From the comparative expression analysis of PtCKXs, OsCKXs, ZmCKXs, and AtCKXs, it was found that tissue-specific gene expression pattern was conserved in each phylogenetic group (Gu et al., 2010). The expression data for *CKX* genes for different studies in *Triticum* were studied from a wheat expression browser. In reproductive stages grain tissue shows expression of *CKX1, CKX2, CKX3, CKX4, CKX5, CKX8, CKX9,* and *CKX11* while *CKX7* And *CKX10* show very low-level expression in the majority of the studies. In leaf and shoot tissue under all three stages, namely*,* reproductive, seedling, and vegetative, we found a good level of expression of *CKX3, CKX4, CKX5, CKX8, CKX9,* and *CKX1*1 while *CKX1* and *CKX*2 had a lower level of expression. *CKX7* and *CKX 10* were found to have the least expression in shoot and leaf tissues in all three developmental stages for the majority of the studies. In root tissues, *CKX1, CKX3, CKX4, CKX5, CKX8,* and *CKX1*1 showed a good level of expression while *CKX9* and *CKX7* had a low level of expression while *CKX2* and *CKX1*0 have the lowest level of expression in reproductive, seedling, and vegetative stages for the majority of the samples for studies reported in wheat expression data. In spike tissue at the reproductive stage, *CKX7* and *CKX10* have very low expression while the rest of *CKX* have a good level of expression. Chen et al., 2020a reported the higher expression of *CKX1* and *CKX2* during grain development similar to our results. Overall, CKX7 expression was at lower levels in the leaf, inflorescence, and spike tissue. CKX4 and CKX5 had a better level of expression in the leaf tissue. In all the tissues studied, CKX3 and CKX11 were expressed at various levels while the expression of CKX10 was found at lower level in all tissues except leaves. These expression trends across tissues found in our study are in concordance with the qRT-PCR results of wheat (Ogonowska et al., 2019). CKX genes functionally diverged and expanded after duplication in angiosperm having tissue/organ-specific patterns that show abiotic stimulus response of expression (Wang et al., 2020).

Gene ontology analysis shows the enrichment of the biological processes, cytokinin metabolic process, cellular hormone metabolic process, hormone metabolic process, and regulation of hormone levels (Supplementary Figure S2). The molecular functions enriched were cytokinin dehydrogenase activity, oxidoreductase activity, acting on the CH-NH group of donors, and flavin adenine dinucleotide binding (Supplementary Figure S3). Gene ontology analysis shows the enrichment of cellular hormone metabolic process, regulation of hormone levels, and molecular functions like oxidoreductase activity and flavin adenine dinucleotide binding were enriched which shows the role of *CKX* in regulating developmental events, enhancing grain yield in rice and enhancing drought and heat stress tolerance in tobacco (Mackova et al.,2013; Werner et al., 2010).

## Conclusion

In this study, we identified 31 members of the *CKX* family in *Triticum* and analyzed their distribution on chromosome, subcellular localization, molecular weight, theoretical isoelectric point, signal peptide, motifs, domains, gene structure, cis-elements, and phylogenetic relationship among *CKX* members. The highest number of phytohormone-responsive cis-elements was found in the *CKX* gene, so, the protomer region has elements involved in the development and growth of *Triticum. CKX*7 and *CKX10* were found to have very low gene expression compared to other *CKX* subgene family members in both reproductive and vegetative stages of *Triticum*. The *CKX* expression from the wheat express database and the analysis at vegetative and reproductive stages in different tissues showed that *CKX* has tissue-specific expression. Gene ontology analysis showed the enrichment of cellular hormone metabolic process, regulation of hormone levels, and molecular functions like oxidoreductase activity and flavin adenine dinucleotide binding that obviates the role of *CKX* in regulating developmental events, and enhancing grain yield and stress tolerance. This study gives a platform for the further identification of the *CKX* family and increases our understanding about the role of *CKX* in the regulation of biotic and abiotic stress resistance, growth, and development in *Triticum* to endeavor for higher production and proper management.

## Data Availability

The original contributions presented in the study are included in the article/Supplementary Material; further inquiries can be directed to the corresponding author.
