# Supplementary material for: Genome-Wide Analysis and Evolutionary Perspective of the Cytokinin Dehydrogenase Gene Family in Wheat (Triticum aestivum L.)
Source: Front Genet. 2022 Aug 19;13:931659. doi: 10.3389/fgene.2022.931659 (PMC9437647; doi:10.3389/fgene.2022.931659)
Supplement: Supplementary file 1 [file Table1.docx]

**Supplementary Table**

**Supplementary Table 1A: Presence of N-terminal presequences: signal peptide (SP), mitochondrial transit peptide (mTP), chloroplast transit peptide (cTP) or thylakoid luminal transit peptide (lTP) in cytokinin dehydrogenase (*Triticum aestivum*) proteins through TargetP-2.0**

| **ID** | **Prediction** | **OTHER** | **SP** | **mTP** | **cTP** | **lTP** | **CS Position** |
| --- | --- | --- | --- | --- | --- | --- | --- |
| CKX1_3A | SP | 0.00 | 1.00 | 0.00 | 0.00 | 0.00 | CS pos: 24-25. AHG-QT. Pr: 0.8147 |
| CKX1_3B | SP | 0.00 | 1.00 | 0.00 | 0.00 | 0.00 | CS pos: 24-25. AHG-QT. Pr: 0.8146 |
| CKX1_3D | SP | 0.00 | 1.00 | 0.00 | 0.00 | 0.00 | CS pos: 24-25. AHG-QT. Pr: 0.7390 |
| CKX2.1_3A | SP | 0.04 | 0.95 | 0.01 | 0.00 | 0.00 | CS pos: 30-31. TTT-SP. Pr: 0.5765 |
| CKX2.1_3B | OTHER | 0.96 | 0.04 | 0.00 | 0.00 | 0.00 |  |
| CKX2.1_3D | SP | 0.00 | 1.00 | 0.00 | 0.00 | 0.00 | CS pos: 29-30. ATS-AY. Pr: 0.2741 |
| CKX2.2.1_3A | SP | 0.00 | 1.00 | 0.00 | 0.00 | 0.00 | CS pos: 22-23. ARA-HD. Pr: 0.8997 |
| CKX2.2.1_3B | SP | 0.00 | 1.00 | 0.00 | 0.00 | 0.00 | CS pos: 18-19. VQA-AR. Pr: 0.6018 |
| CKX2.2.1_3D | SP | 0.01 | 0.99 | 0.00 | 0.00 | 0.00 | CS pos: 21-22. ARA-DA. Pr: 0.5245 |
| CKX2.2.2_3D | SP | 0.00 | 1.00 | 0.00 | 0.00 | 0.00 | CS pos: 21-22. ARA-DA. Pr: 0.7540 |
| CKX2.2.3_3D | OTHER | 1.00 | 0.00 | 0.00 | 0.00 | 0.00 |  |
| CKX3_1A | SP | 0.11 | 0.88 | 0.00 | 0.00 | 0.00 | CS pos: 27-28. FIQ-SP. Pr: 0.2173 |
| CKX3_1B | SP | 0.13 | 0.86 | 0.00 | 0.00 | 0.01 | CS pos: 27-28. FIQ-SP. Pr: 0.2170 |
| CKX3_1D | SP | 0.13 | 0.86 | 0.00 | 0.00 | 0.01 | CS pos: 27-28. FIQ-SP. Pr: 0.2171 |
| CKX4_3B | SP | 0.00 | 1.00 | 0.00 | 0.00 | 0.00 | CS pos: 23-24. VTM-HV. Pr: 0.3673 |
| CKX4_3D | SP | 0.00 | 1.00 | 0.00 | 0.00 | 0.00 | CS pos: 23-24. VTM-HV. Pr: 0.4034 |
| CKX5_3A | SP | 0.00 | 1.00 | 0.00 | 0.00 | 0.00 | CS pos: 20-21. TVG-LP. Pr: 0.4791 |
| CKX5_3B | SP | 0.00 | 1.00 | 0.00 | 0.00 | 0.00 | CS pos: 20-21. TVG-LP. Pr: 0.4797 |
| CKX5_3D | SP | 0.01 | 0.99 | 0.00 | 0.00 | 0.00 | CS pos: 20-21. TVG-LP. Pr: 0.4789 |
| CKX7_6B | SP | 0.00 | 0.92 | 0.06 | 0.01 | 0.00 | CS pos: 22-23. VAG-QL. Pr: 0.6535 |
| CKX7_6D | SP | 0.00 | 0.93 | 0.06 | 0.01 | 0.00 | CS pos: 22-23. VAG-QL. Pr: 0.5731 |
| CKX8_2A | SP | 0.00 | 1.00 | 0.00 | 0.00 | 0.00 | CS pos: 22-23. AAT-FI. Pr: 0.2391 |
| CKX8_2B | SP | 0.00 | 1.00 | 0.00 | 0.00 | 0.00 | CS pos: 22-23. AAT-FI. Pr: 0.2155 |
| CKX9_1B | SP | 0.00 | 1.00 | 0.00 | 0.00 | 0.00 | CS pos: 22-23. VTT-EH. Pr: 0.8689 |
| CKX9_1D | SP | 0.00 | 1.00 | 0.00 | 0.00 | 0.00 | CS pos: 22-23. VTT-EH. Pr: 0.8689 |
| CKX10_7A | OTHER | 0.52 | 0.23 | 0.00 | 0.09 | 0.15 |  |
| CKX10_7B | SP | 0.06 | 0.93 | 0.01 | 0.00 | 0.00 | CS pos: 36-37. AFG-AL. Pr: 0.4689 |
| CKX10_7D | SP | 0.01 | 0.99 | 0.00 | 0.00 | 0.00 | CS pos: 28-29. AFG-AL. Pr: 0.5221 |
| CKX11_7A | OTHER | 1.00 | 0.00 | 0.00 | 0.00 | 0.00 |  |
| CKX11_7B | OTHER | 1.00 | 0.00 | 0.00 | 0.00 | 0.00 |  |
| CKX11_7D | OTHER | 1.00 | 0.00 | 0.00 | 0.00 | 0.00 |  |

**Supplementary Table 1B: Signal peptides and the location of their cleavage sites in Cytokinin Dehydrogenase (*Triticum aestivum*) proteins through SignalP-6.0**

| **# ID** | **Prediction** | **OTHER** | **SP(Sec/SPI)** | **CS Position** |
| --- | --- | --- | --- | --- |
| CKX1_3A | SP | 0.00 | 1.00 | CS pos: 24-25. Pr: 0.9056 |
| CKX1_3B | SP | 0.00 | 1.00 | CS pos: 24-25. Pr: 0.9056 |
| CKX1_3D | SP | 0.00 | 1.00 | CS pos: 24-25. Pr: 0.8532 |
| CKX2.1_3A | SP | 0.02 | 0.98 | CS pos: 36-37. Pr: 0.6429 |
| CKX2.1_3B | NO_SP | 0.93 | 0.07 |  |
| CKX2.1_3D | SP | 0.00 | 1.00 | CS pos: 30-31. Pr: 0.6737 |
| CKX2.2.1_3A | SP | 0.00 | 1.00 | CS pos: 22-23. Pr: 0.9431 |
| CKX2.2.1_3B | SP | 0.00 | 1.00 | CS pos: 18-19. Pr: 0.9803 |
| CKX2.2.1_3D | SP | 0.00 | 1.00 | CS pos: 18-19. Pr: 0.9823 |
| CKX2.2.2_3D | SP | 0.00 | 1.00 | CS pos: 21-22. Pr: 0.9355 |
| CKX2.2.3_3D | NO_SP | 1.00 | 0.00 |  |
| CKX3_1A | NO_SP | 0.71 | 0.29 |  |
| CKX3_1B | NO_SP | 0.71 | 0.29 |  |
| CKX3_1D | NO_SP | 0.71 | 0.29 |  |
| CKX4_3B | SP | 0.00 | 1.00 | CS pos: 23-24. Pr: 0.9302 |
| CKX4_3D | SP | 0.00 | 1.00 | CS pos: 23-24. Pr: 0.9448 |
| CKX5_3A | SP | 0.00 | 1.00 | CS pos: 20-21. Pr: 0.9819 |
| CKX5_3B | SP | 0.00 | 1.00 | CS pos: 20-21. Pr: 0.9819 |
| CKX5_3D | SP | 0.00 | 1.00 | CS pos: 20-21. Pr: 0.9819 |
| CKX7_6B | SP | 0.00 | 1.00 | CS pos: 22-23. Pr: 0.9547 |
| CKX7_6D | SP | 0.00 | 1.00 | CS pos: 22-23. Pr: 0.9563 |
| CKX8_2A | SP | 0.22 | 0.78 | CS pos: 20-21. Pr: 0.7504 |
| CKX8_2B | SP | 0.26 | 0.74 | CS pos: 20-21. Pr: 0.7117 |
| CKX9_1B | SP | 0.00 | 1.00 | CS pos: 22-23. Pr: 0.9670 |
| CKX9_1D | SP | 0.00 | 1.00 | CS pos: 22-23. Pr: 0.9670 |
| CKX10_7A | NO_SP | 1.00 | 0.00 |  |
| CKX10_7B | SP | 0.14 | 0.86 | CS pos: 36-37. Pr: 0.6826 |
| CKX10_7D | NO_SP | 0.52 | 0.48 |  |
| CKX11_7A | NO_SP | 1.00 | 0.00 |  |
| CKX11_7B | NO_SP | 1.00 | 0.00 |  |
| CKX11_7D | NO_SP | 1.00 | 0.00 |  |

**Supplementary Table 2: CKS synteny and collinearity in chromosomes of *Triticum astevium* and *Aegilops tauschii***

**Supplementary Table 3: Different number of Cis-element under *CKX***

| **Cis elements** | **LTR** | **MBS** | **MRE** | **Sp1** | **GT1-motif** | **ACE** | **TC-rich repeats** | **TCA-element** | **ABRE** | **GARE-motif** | **TGA-element** | **AuxRR-core** | **TATC-box** | **P-box** |
| --- | --- | --- | --- | --- | --- | --- | --- | --- | --- | --- | --- | --- | --- | --- |
| **Role of cis- element** | **cis-acting element involved in low-temperature responsiveness** | **MYB binding site involved in drought-inducibility** | **MYB binding site involved in light responsiveness** | **light responsive element** | **light responsive element** | **cis-acting element involved in light responsiveness** | **cis-acting element involved in defense and stress responsiveness** | **cis-acting element involved in salicylic acid responsiveness** | **cis-acting element involved in the abscisic acid responsiveness** | **gibberellin-responsive element** | **auxin-responsive element** | **cis-acting regulatory element involved in auxin responsiveness** | **cis-acting element involved in gibberellin-responsiveness** | **gibberellin-responsive element** |
| **CKX1_3A** | 1 | - | - | - | - | - | - | 1 | - | - | - | - | - | - |
| **CKX1_3B** | 1 | 1 | 1 | - | - | - | - | 1 | 1 | - | - | - | - | - |
| **CKX1_3D** | - | - | 1 | - | - | - | - | - | 1 | 1 | - | - | - | - |
| **CKX2.1_3A** | - | - | - | 1 | - | - | - | 1 | 1 | - | - | - | - | 1 |
| **CKX2.1_3B** | 1 | 1 | - | - | - | - | 1 | - | 1 | - | - | - | - | - |
| **CKX2.1_3D** | 1 | 1 | - | 1 | - | - | 1 | - | 1 | - | 1 | - | - | - |
| **CKX2.2.1_3A** | - | - | - | 1 | - | - | - | - | 1 | - | - | - | - | - |
| **CKX2.2.1_3B** | 1 | - | - | - | - | - | 1 | - | 1 | 1 | 1 | - | - | 1 |
| **CKX2.2.1_3D** | 1 | - | - | - | 1 | - | - | - | 1 | - | - | - | - | - |
| **CKX2.2.2_3D** | 1 | - | - | 1 | - | - | - | - | 1 | - | - | - | - | - |
| **CKX2.2.3_3D** | - | 1 | 1 | - | 1 | - | - | - | 1 | - | 1 | - | - | 1 |
| **CKX3_1A** | 1 | 1 | - | 1 | - | - | - | 1 | - | - | 1 | 1 | - | 1 |
| **CKX3_1B** | 1 | 1 | - | - | - | - | - | - | 1 | - | - | - | - | 1 |
| **CKX3_1D** | 1 | - | - | - | - | - | - | 1 | 1 | - | - | - | - | 1 |
| **CKX4_3B** | - | 1 | - | 1 | 1 | - | - | - | 1 | - | - | - | - | - |
| **CKX4_3D** | - | 1 | - | 1 | 1 | - | - | - | 1 | - | 1 | - | - | - |
| **CKX5_3A** | - | - | - | 1 | - | - | - | - | 1 | 1 | - | - | - | - |
| **CKX5_3B** | - | 1 | - | 1 | 1 | 1 | - | - | 1 | - | - | - | 1 | - |
| **CKX5_3D** | - | 1 | - | 1 | 1 | 1 | - | - | 1 | - | - | - | - | - |
| **CKX7_6B** | 1 | - | - | - | - | 1 | - | - | 1 | - | 1 | - | 1 | - |
| **CKX7_6D** | - | - | - | 1 | 1 | - | - | 1 | 1 | - | 1 | 1 | - | - |
| **CKX8_2A** | - | - | - | 1 | - | - | - | - | 1 | 1 | - | - | - | - |
| **CKX8_2B** | - | - | - | 1 | - | - | - | - | 1 | 1 | - | - | - | - |
| **CKX9_1B** | - | 1 | - | - | - | - | 1 | - | 1 | - | - | - | - | - |
| **CKX9_1D** | - | 1 | - | - | - | 1 | 1 | - | 1 | - | - | - | - | - |
| **CKX1-_7A** | 1 | - | - | 1 | - | - | - | - | 1 | - | 1 | - | - | - |
| **CKX1-_7B** | - | - | 1 | - | - | - | - | 1 | - | 1 | - | - | - | - |
| **CKX1-_7D** | - | - | 1 | 1 | - | - | - | 1 | 1 | 1 | - | 1 | - | - |
| **CKX11_7A** | - | - | - | - | - | - | - | - | - | - | - | - | - | - |
| **CKX11_7B** | - | - | - | 1 | - | - | - | - | 1 | - | 1 | 1 | - | - |
| **CKX11_7D** | - | - | - | 1 | - | - | - | - | 1 | - | 1 | 1 | - | - |
