# Supplementary figures and images for: Genome-Wide Analysis and Evolutionary Perspective of the Cytokinin Dehydrogenase Gene Family in Wheat (Triticum aestivum L.)
Source: Front Genet. 2022 Aug 19;13:931659. doi: 10.3389/fgene.2022.931659 (PMC9437647; doi:10.3389/fgene.2022.931659)

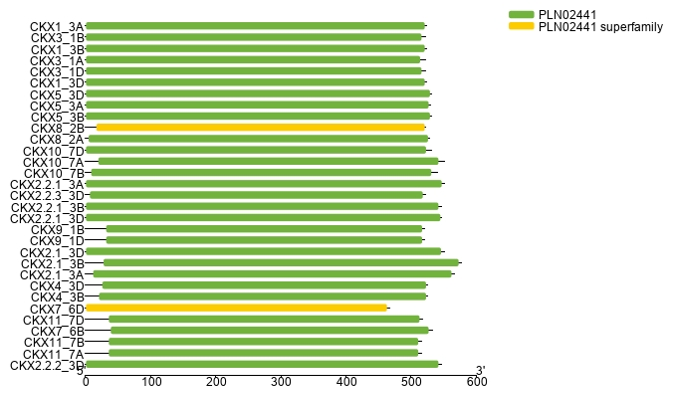

Supplement: Supplementary file 2 [file Image1.TIF]

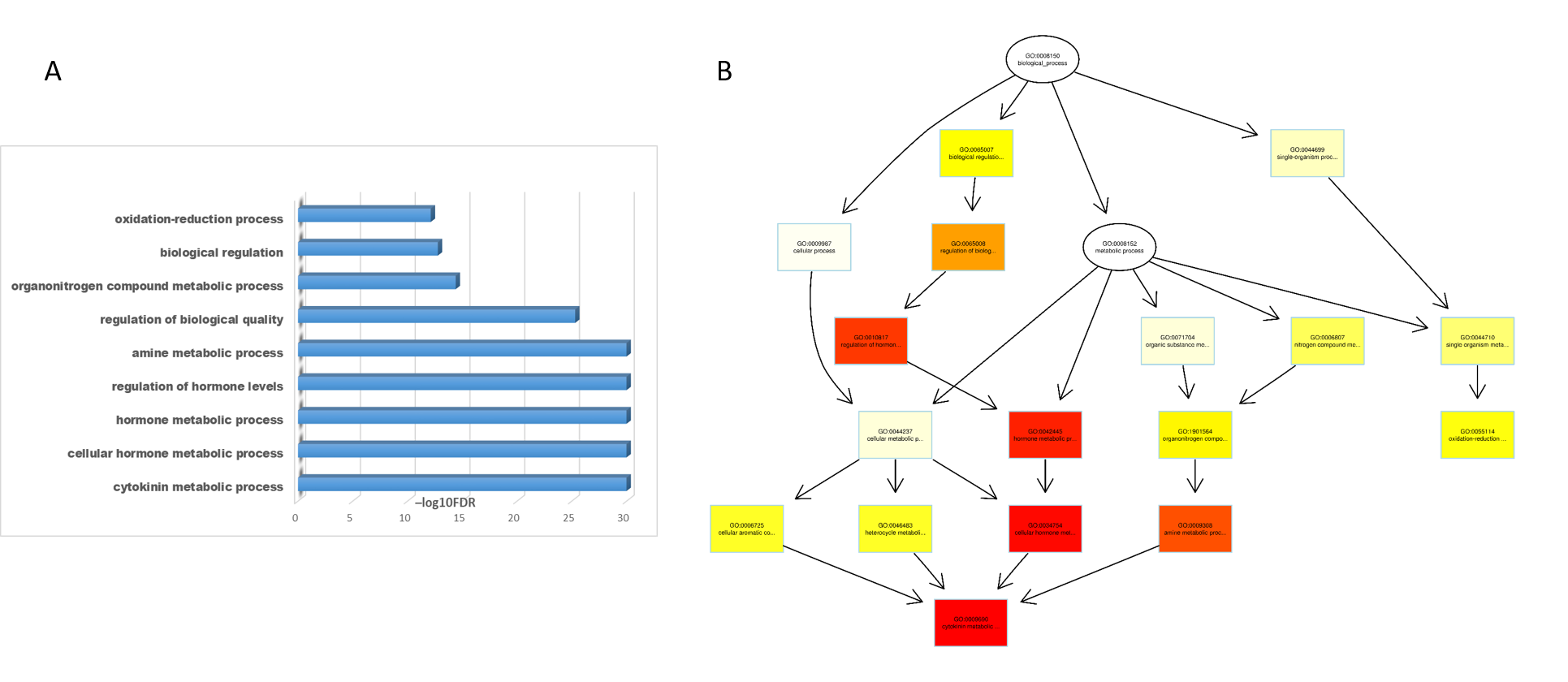

Supplement: Supplementary file 3 [file Image2.PNG]

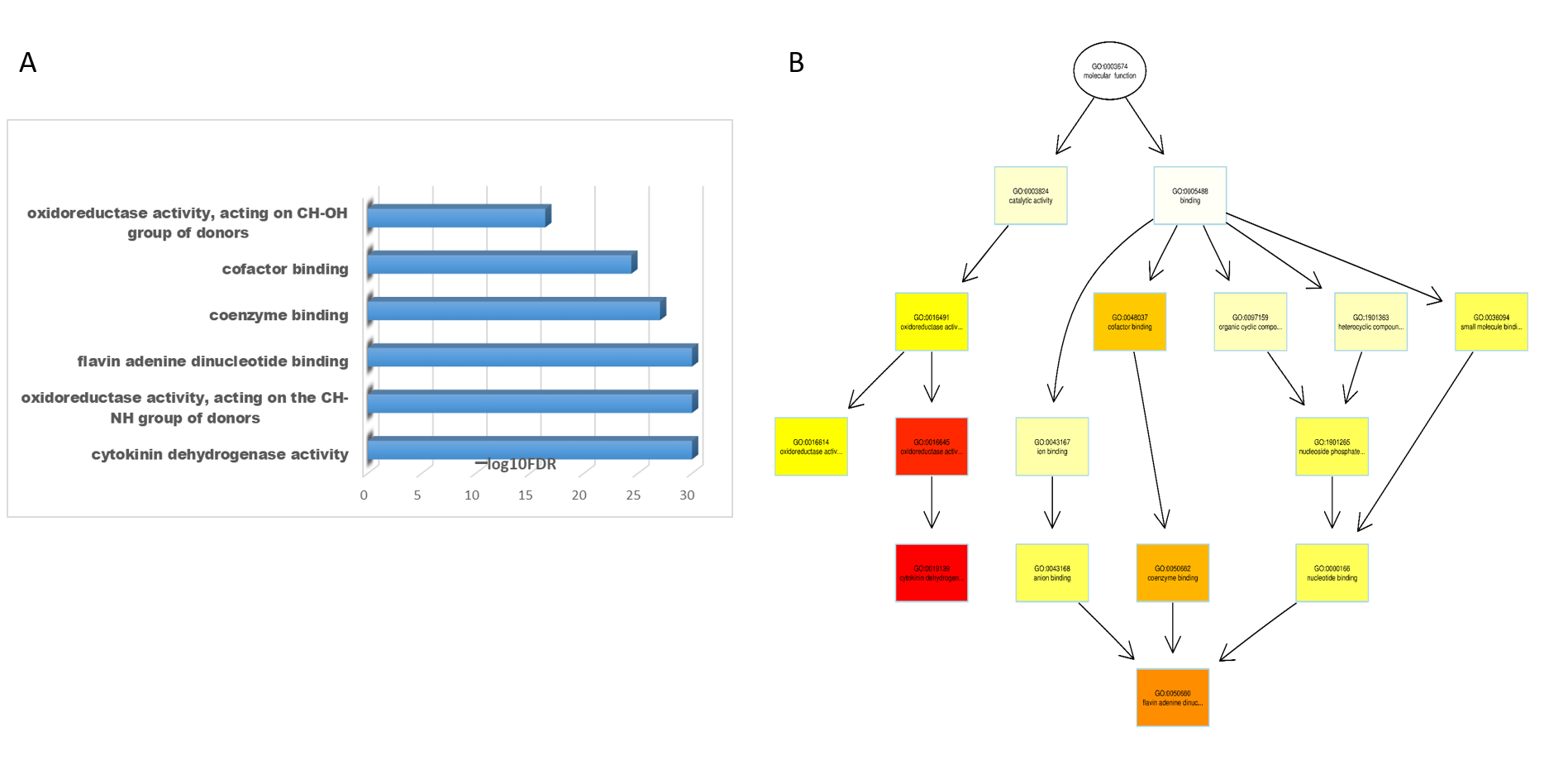

Supplement: Supplementary file 4 [file Image3.PNG]
